# Supplementary figures and images for: N-acetylcysteine alleviates PCB52-induced hepatotoxicity by repressing oxidative stress and inflammatory responses
Source: PeerJ. 2020 Aug 11;8:e9720. doi: 10.7717/peerj.9720 (PMC7427542; doi:10.7717/peerj.9720)

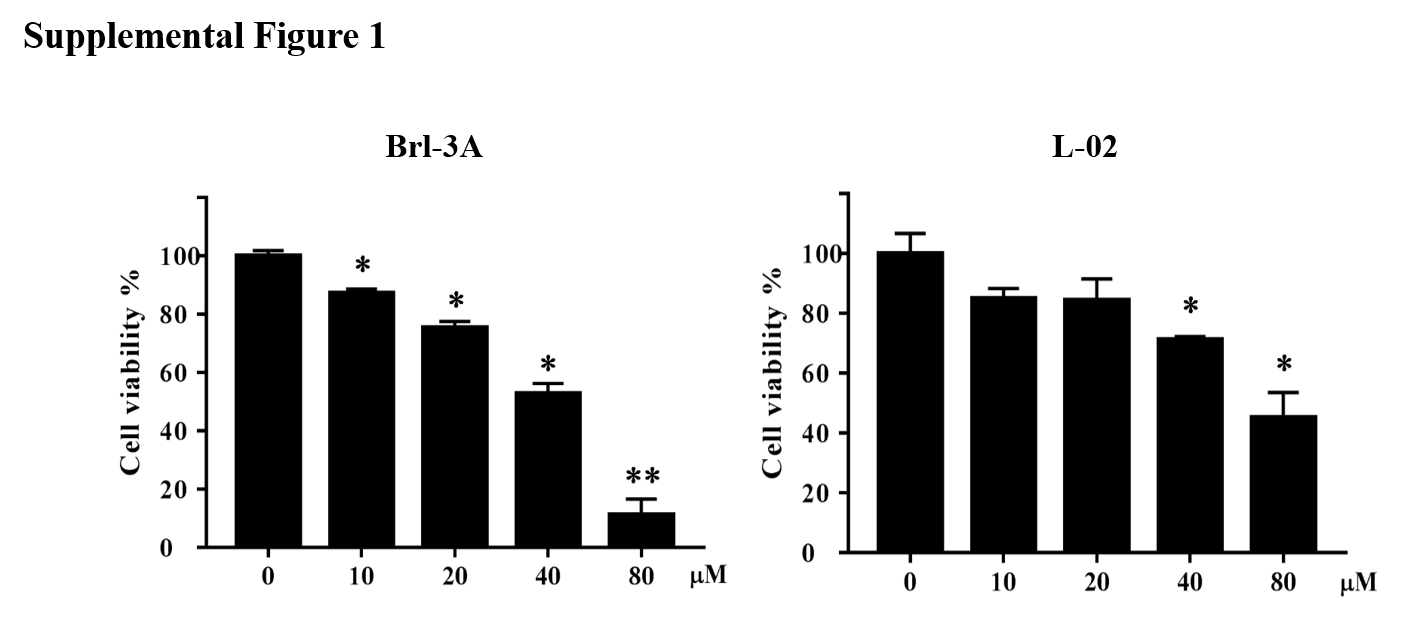

Supplement: Supplemental Information 1 — PCB52 dose-dependently decreased cell viabilities in both Brl-3A and L-02 cells, especially at 40 and 80 μM. *p < 0.05, **p < 0.01, relative to the control. [file peerj-08-9720-s001.png]

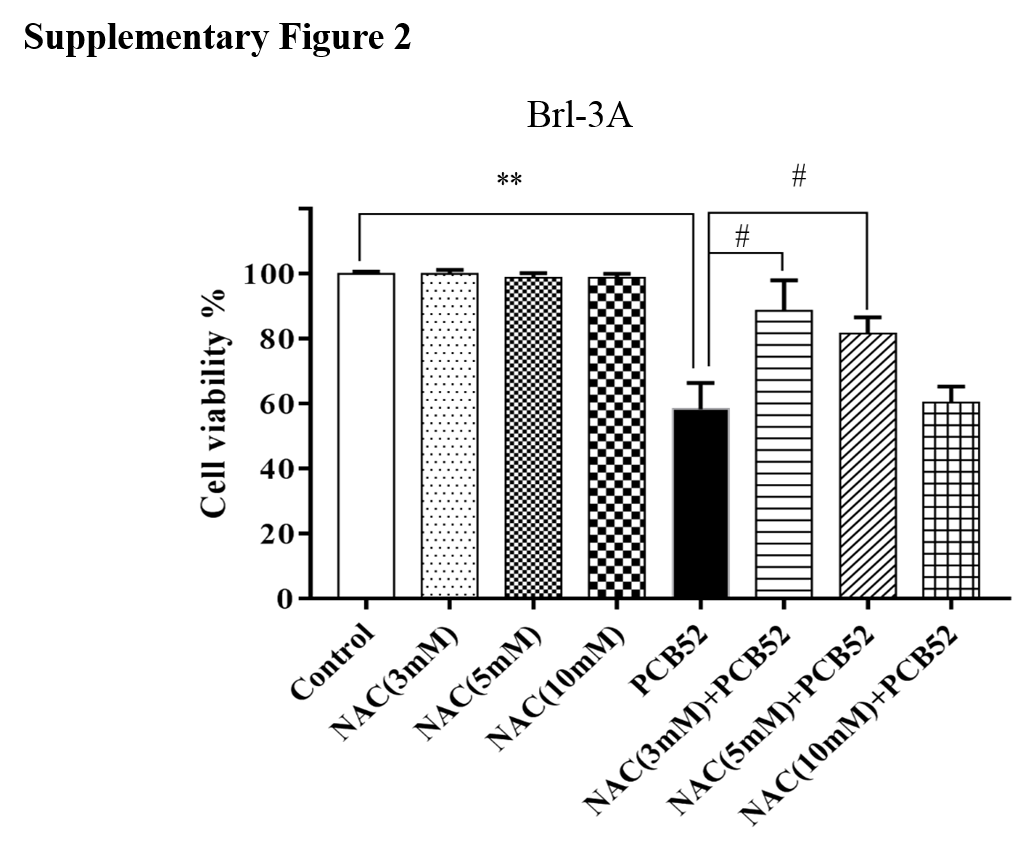

Supplement: Supplemental Information 2 — No changes of cell viability were observe in NAC alone treatment groups compared with the control. Cell viability was significantly reduced by PCB52. The pretreatment of NAC (3 and 5 mM) significantly alleviated PCB52-induced cytotoxicity. **p < 0.01, compared with the control; #p < 0.05, compared with the PCB52 group. [file peerj-08-9720-s002.png]
