# Supplementary material for: N-acetylcysteine alleviates PCB52-induced hepatotoxicity by repressing oxidative stress and inflammatory responses
Source: PeerJ. 2020 Aug 11;8:e9720. doi: 10.7717/peerj.9720 (PMC7427542; doi:10.7717/peerj.9720)
Supplement: Supplemental Information 3 [file peerj-08-9720-s003.docx]

Supplementary Table 1. Primers for RT-qPCR analysis

| Gene | F (5’–3’) | R (5’–3’) |
| --- | --- | --- |
| Rat |  |  |
| Traf6 | ATGAAGAGAAAGAGATCCACGA | CTCTTATGAGGATTGTACCACAG |
| Myd88 | GTTTCTGACGATTACCTGCA | TTCTGTTGGACACCTGGAG |
| Tnf | CTTCTCATTCCTGCTCGTG | TTTGGGAACTTCTCCTCCT |
| Keap1 | TGCTCAACCGCTTGCTGTAT | ATCATCCGCCACTCATTCCT |
| Nfe2l2 | ATTGCTGTCCATCTCTGTCAG | GCTATTTTCCATTCCCGAGTTAC |
| Hmox1 | GCCTGGCACATTTCCCTCAC | CAGAACAGCCGCCTCTACCG |
| Nqo1 | GAGAGTGCTTGTAGCAGGA | AGCAAGGTCTTCTTATTCTGGA |
| Sqstm1 | AGAATGTGGGGGAGAGCGTGGC | GGGTGTCAGGCGGCTTCTCTT |
| β-actin | CGTGAAAAGATGACCCAGATCA | AGAGGCATACAGGGACAACACA |
| Human |  |  |
| Traf6 | CCATGACCAGAACTGTCCT | ATGATTAGGCATCTGTTCTCTG |
| MYD88 | CAGCATTGAGGAGGATTGC | GGGACACTGCTGTCTACAG |
| TNF | CTCTAATCAGCCCTCTGGC | GAGGGTTTGCTACAACATGG |
| IL1B | GCTTATTACAGTGGCAATGAGG | AGATTCGTAGCTGGATGCC |
| KEAP1 | CGGGAGTACATCTACATGC | GACAGGTTGAAGAACTCCTC |
| NFE2L2 | CCAGTCAGAAACCAGTGGA | ATCTGATTTGGGAATGTGGG |
| HMOX1 | AACTCCCTGGAGATGACTC | CTCAAAGAGCTGGATGTTGA |
| NQO1 | ACATCACAGGTAAACTGAAGG | TCAGATGGCCTTCTTTATAAGC |
| β-actin | TCCCTGGAGAAGAGCTACGA | AGCACTGTGTTGGCGTACAG |
